# The global diet and activity research (GDAR) network: a global public health partnership to address upstream NCD risk factors in urban low and middle-income contexts

**DOI:** 10.1186/s12992-020-00630-y

**Published:** 2020-10-19

**Authors:** Tolu Oni, Felix Assah, Agnes Erzse, Louise Foley, Ishtar Govia, Karen J. Hofman, Estelle Victoria Lambert, Lisa K. Micklesfield, Maylene Shung-King, Joanne Smith, Eleanor Turner-Moss, Nigel Unwin, Pamela Wadende, James Woodcock, Jean Claude Mbanya, Shane A. Norris, Charles O. Obonyo, Marshall Tulloch-Reid, Nicholas J. Wareham, on behalf of the GDAR network, Nadia Bennett, Anna Brugulat, Nathalie Guthrie-Dixon, Ian Hambleton, Kelsey Lebar, Gugulethu Mabena, Clarisse Mapa, Ebele Mogo, Camille Mba, Molebogeng Motlhalhedi, Rosemary Musuva, Feyisayo A. Odunitan-Wayas, Kufre J. Okop, Lambed Tatah, Yves Wasnyo, Amy Weimann, Vincent Were

**Affiliations:** 1 MRC Epidemiology Unit, Institute of Metabolic Sciences Building, Addenbrookes Hospital, University of Cambridge, Cambridge, CB2 0QQ UK; 2 Research Initiative for Cities Health and Equity (RICHE), School of Public Health and Family Medicine, University of Cape Town, Cape Town, South Africa; 3 Health of Populations in Transition (HoPiT), Research Group, Faculty of Medicine and Biomedical Sciences, The University of Yaoundé I, Yaoundé, Cameroon; 4 SA MRC Centre for Health Economics and Decision Science (PRICELESS SA), Faculty of Health Sciences, School of Public Health, University of Witwatersrand, Johannesburg, South Africa; 5 Caribbean Institute for Health Research, The University of West Indies, Kingston, Jamaica; 6 Health through Physical Activity Lifestyle and Sport Research Centre, University of Cape Town, Cape Town, South Africa; 7 MRC/Wits Developmental Pathways for Health Research Unit (DPHRU), University of Witwatersrand, Johannesburg, South Africa; 8 Centre for Global Health Research, Kenya Medical Research Institute, Kisumu, Kenya

**Keywords:** Upstream determinants, Non-communicable diseases, Diet, Physical activity, Partnerships, Global health, LMICs

## Abstract

**Background:**

Non-communicable diseases (NCDs) are the leading cause of death globally. While upstream approaches to tackle NCD risk factors of poor quality diets and physical inactivity have been trialled in high income countries (HICs), there is little evidence from low and middle-income countries (LMICs) that bear a disproportionate NCD burden. Sub-Saharan Africa and the Caribbean are therefore the focus regions for a novel global health partnership to address upstream determinants of NCDs.

**Partnership:**

The Global Diet and Activity research Network (GDAR Network) was formed in July 2017 with funding from the UK National Institute for Health Research (NIHR) Global Health Research Units and Groups Programme. We describe the GDAR Network as a case example and a potential model for research generation and capacity strengthening for others committed to addressing the upstream determinants of NCDs in LMICs. We highlight the dual equity targets of research generation and capacity strengthening in the description of the four work packages. The work packages focus on *learning from the past* through identifying evidence and policy gaps and priorities, *understanding the present* through adolescent lived experiences of healthy eating and physical activity, and *co-designing future interventions* with non-academic stakeholders.

**Conclusion:**

We present five lessons learned to date from the GDAR Network activities that can benefit other global health research partnerships. We close with a summary of the GDAR Network contribution to cultivating sustainable capacity strengthening and cutting-edge policy-relevant research as a beacon to exemplify the need for such collaborative groups.

## Background

Many low and middle-income countries (LMICs) are experiencing rapid, unplanned urbanization, resulting in a significant proportion of urban dwellers living in informal settlements, exposed to worse conditions than in rural areas [1] and to multiple non-communicable disease (NCD) risk factors including poor quality diets and insufficient physical activity. Upstream approaches to NCD prevention in LMICs therefore need to consider urban exposures and the connection between relevant health indicators and urban development initiatives [2]. However, there is limited context-specific and local evidence from LMICs to inform policy responses at the neighbourhood, city, metropolitan or national level. With the majority of urban residents in LMICs simultaneously experiencing political, economic, housing and ecological vulnerability [3], research into the impact of interventions targeting urban spaces, such as the food and built environment on health behaviours and disease outcomes in the long-term can be transformative for addressing health and social inequity [2].

Dealing with upstream determinants of the rising burden of NCD needs engagement with the socially determined drivers of NCD risk factors, as well as the structural inequities in knowledge creation. Such evidence needs to be generated by research that incorporates an understanding of locally relevant policy, political, historical, economic and social contexts. Furthermore, such research requires collaboration across disciplines and geographies to provide opportunities for learning from similar settings and for mutually beneficial resource exchanges.

The expectations of global partnerships are changing with acknowledgment of the importance of joint agenda setting, bidirectional shared learning [4] and collective decision making. However, this evolving narrative can mask persistent inequities in the nature of global partnerships [5]. The concept of “reverse innovation” has been used to describe the notion that innovations from low and middle-income countries might be transferable to high-income contexts [6]. But there is bias embedded in this term with the expectation that innovation would “normally” flow from high to LMICs, with reverse innovation somehow being opposite to this expectation. This interpretation ignores historical accounts of health innovations from the global south which have been of global benefit such as the use of variolation which was long practiced in Africa and Asia before learned by Europeans to combat smallpox. Similarly the use of artemisinin was developed from Chinese medicine for malaria control and the Qanun fi-l-tibb (Canon of Medicine) of Ibn Sina was an encyclopaedia of medicine that informed medical science in Europe for over 600 years [7].

A bibliometric analysis of priority interventions for NCDs in LMICs found that less than a quarter of publications between 2000 and 2011 were related to either physical activity or nutrition [8]. However, fewer than 10% of the total number of publications used evidence specifically from Latin America, the Caribbean and Sub-Saharan Africa, combined. In those cases where there was data from these regions, just over half of the papers had lead authors from the region [8]. Another review found that 38% of research published in LMIC focused on NCDs [9]. Regional authorship is important, as it provides local interpretations and insights to the evidence and an opportunity to translate that evidence to inform policies and programmes, that are locally relevant and culturally salient [10]. We sought to develop the GDAR partnership body of work cognisant of, and confronting, these persisting biases and inequities.

Here we describe the GDAR Network as a case example and a potential model for research generation and capacity building for others committed to addressing the upstream determinants of NCDs in LMICs. A key component of this work entails stakeholder engagement to understand the evidence and policy gaps and priorities for each setting. These gaps informed the research agenda that was collaboratively set by the network members. Contextual factors were considered and incorporated into the research design and methods.

## The Global Diet and Activity Research (GDAR) network

The Global Diet and Activity Research Network (GDAR) (https://www.gdarnet.org/) was formed in July 2017 with funding from the United Kingdom (UK) National Institute for Health Research (NIHR) Global Health Research Units and Groups Programme (https://www.nihr.ac.uk/explore-nihr/funding-programmes/global-health/) granted to University of Cambridge’s MRC Epidemiology Unit to contribute to addressing the health needs of populations in LMICs in partnership with investigators in 3 countries in sub-Saharan Africa and one in the Caribbean: Cameroon, Kenya, South Africa and Jamaica respectively. The health need of focus in these LMICs is the prevention of NCDs, including cardiovascular diseases, type 2 diabetes, and cancers in LMICs.

### Collaborative research agenda setting

**Fig. 1:**
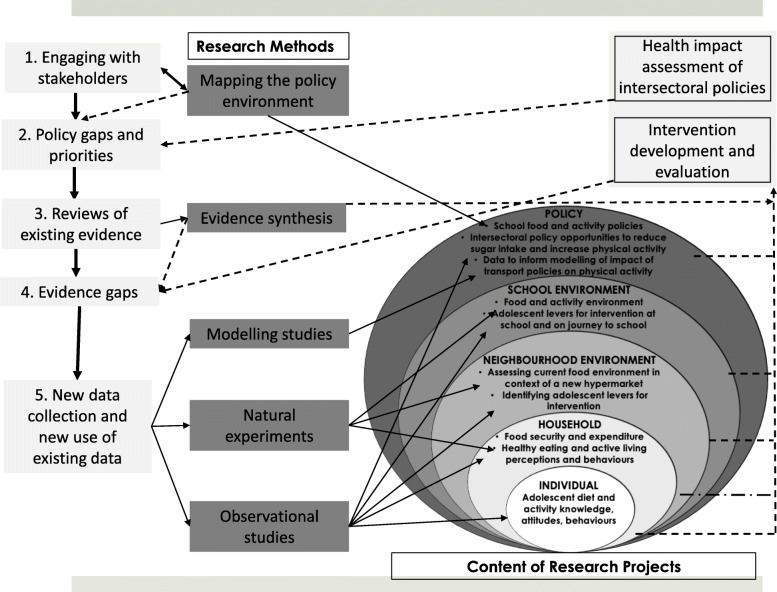
Conceptual framework for policy-relevant impactful research in the GDAR network. The process began with engaging stakeholders to identify policy and evidence gaps. This co-design process influenced the choice of research methods and nature of research conducted at varying socio-ecological levels from individual to policy. Dotted lines indicate desired pathways to impact to address evidence gaps

A critical factor in the success of any such initiative is collaborative research partnership governance and agenda setting. Figure 1 illustrates the components of the GDAR Network’s research agenda jointly developed between the MRC Epidemiology Unit and the partner institutions in each of the LMICs (two in South Africa and one in each of the other countries). The GDAR Network objective is to generate evidence on the upstream determinants of diet and physical activity (PA) in Africa and the Caribbean (Fig. 1). GDAR Network partner institutions identified that sub-Saharan African and Caribbean countries face common food system and built environment challenges. In a two-day face-to-face workshop in 2017 in the UK and in 2018 in South Africa, a balance within the research agenda was reached by population, geography, topic and approach.

Research activities are led by institutions and investigators in both LMICs and the UK. Leadership for different aspects of the research, and activities to build capacity, are shared according to expertise across the whole network. A range of participatory transdisciplinary approaches were used to develop the research agenda to explore the role of upstream determinants of diet and activity in Africa and the Caribbean. Research methods have been co-developed with some level of flexibility to accommodate variation between sites as not all variables were available within each country, and specific criteria and methodology were created for cross-country analyses. These collaborative approaches maximize the use of established datasets and research investments. This type of cross-site sharing and learning fosters novel research questions, particularly those not answerable solely from individual site data. The sustained willingness to cooperate across sites is based on the anticipated impact that this research process and outcomes will continue to provide innovative and more relevant insights into best practices of prevention of diet and physical activity related NCD risk factors.

**Fig. 2:**
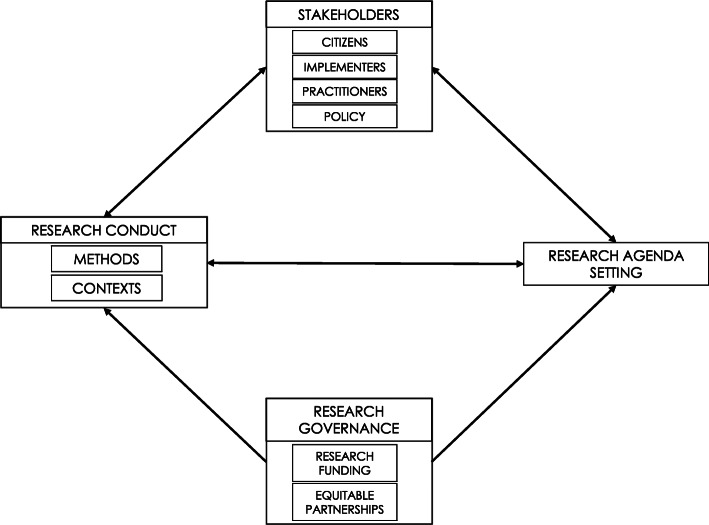
Transdisciplinary and collaborative approaches to agenda setting and research conduct

The priorities for research were set jointly between all partners and informed by consultation with stakeholders from the participating contexts. In-country researchers co-developed and executed the research programmes through the engagement of local and global partners, creating a South-to-South driven research collaboration. Locally designed and driven research is key to ensuring that the research is relevant to contextually specific challenges [11]. It also helps increase local ownership of the interventions and drives uptake into practice. The GDAR Network research agenda has been informed by pragmatic efforts to ‘close the loop’ back to policy-relevant science using the input of relevant stakeholders as contributors of knowledge. Figure 2 illustrates a research agenda setting process driven by equitable research partnerships and stakeholder engagement to inform the conduct of contextual and impactful research.

### GDAR network work packages

To generate evidence on the upstream determinants of diet and PA in Africa and the Caribbean, the GDAR Network designed and implemented four work packages (WPs) (Table 1). During the research prioritisation exercises of the two-day face-to-face investigator workshops, in 2017 in the UK and in 2018 in South Africa, the interlinkages between work packages were emphasised. Before setting the agenda, the network joint lead visited all partner sites seeking input in developing a research agenda to engage issues of diet and PA behaviours in LMICs. During these visits, the joint lead held consultative meetings with investigators in the partner sites where partners shared ongoing work, strengths, and capacity-strengthening requirements. The information presented incorporated experiences from interactions between partner teams and their stakeholders, including experiences of research agenda setting and knowledge translation. Thus, knowledge exchange strategies were built into research protocols from the outset and revisited at every stage.

**Table 1:** GDAR Network Work Packages

Work Packages (WP)	Details
WP1a: Evidence synthesis	WP1a: Systematic reviews on a) factors associated with (active) travel behaviour; and b) the use of social and community networks for physical activity in Africa and the Caribbean
WP1b: Assessing data availability	WP1b: Assessing availability of data on transport behaviours and road traffic injury patterns to inform development of models of the health impact of transport policies in Africa
WP2: Adolescent levers	Adolescent levers for diet and physical activity intervention across socio-ecological levels in 4 low and middle-income countries
WP3: Policy analysis	A multi-level (global, regional, national, sub-national) intersectoral policy space and content analysis of policies that influence food and activity built environments: Implications for low and middle-income countries in Africa and the Caribbean
WP4: Natural experimental studies	WP4a: Impact of a new hypermarket on food purchasing and dietary behaviours in Kisumu, Kenya
	WP4b: Evaluating implementation of a voluntary pledge to remove advertisement of sugar-sweetened beverages around schools in Johannesburg, South Africa

In the sections below, we present the GDAR Work Packages as they correspond to the generation of research to learn from the past, understand the present and co-create the future. While findings are pending with research underway at the time of writing, we share the lessons that we have learned from our experience to date as a global partnership for addressing the upstream determinants of NCDs and fostering related multisectoral approaches.

## Learning from the past: identifying evidence and policy gaps and priorities

### Evidence synthesis (WP1a)

An initial scoping review of reviews was conducted to identify and describe summarised evidence on factors associated with diet and physical inactivity in LMICs in Africa and the Caribbean. We searched for reviews (incorporating quantitative and qualitative data) published in the last 10 years including at least 25% of studies from Africa or the Caribbean. The factors associated with diet and PA from these reviews were charted and categorised as more proximal (e.g. age or sex) or distal (e.g. cultural, environmental or policy) to the individual. We identified 16 relevant reviews. There were notable gaps in the summarised evidence found for Caribbean populations, particularly for the more distal factors. Across all of the levels of influence ranging from proximal to distal, there was a lack of summarised evidence on the factors influencing PA.

The findings from this initial piece of work were used by GDAR researchers to guide the development of two systematic reviews of the primary literature. The first of these explored factors associated with travel behaviour, in particular walking and cycling, in Africa and the Caribbean. The second examined the use of social and community networks and social networking sites for physical activity in Africa and the Caribbean and strategies to increase their effectiveness and feasibility. Together these reviews will address the identified gap of summarised evidence on the distal factors that shape diet and PA in these regions (Fig. 1).

### Policy space and content analysis (WP3)

Policy is fundamental in shaping practice, and practice shapes future policy directions. Policy is significantly shaped by many factors, including context and history. Country policy direction is often shaped by the overall global context and this is very pertinent in the case of NCDs. We therefore identified the need to conduct research to identify and detail a historical timeline of intersectoral policies and policy opportunities, at varying contextual scales from global to subnational, in Africa and the Caribbean. This work package which focuses on policy analysis of global and national policies, retrospectively explored the content of relevant global and local policies on diet and PA, to elicit current policy direction and intentions in these two determinants of NCDs (Fig. 1). For the analysis, we mainly engaged with written policy documents, published as official policy positions, by international agencies across relevant sectors and country governments. Our work focused on exploring how policy on NCDs and its determinants have evolved over a 20-year period, starting with the onset of the Millennium Development Goals in 2000. We furthermore explored how these policies are reinterpreted and made relevant to country contexts. We simultaneously engaged with country-level policy makers in an effort to bring policy analysis findings to bear on the development of locally relevant policies, drawing on global policy proposals, as well as to co-create implementation pathways that are cognisant of policy goals and priorities.

Simultaneously, other GDAR work packages are generating new and novel evidence on how citizens engage with, and experience, aspects of a healthy diet and PA, the access to and availability of which are both directly and indirectly influenced by policy. This could help shape ideas on how to generate locally relevant policy goals and implementation of interventions towards primary prevention of NCDs.

## Understanding the present: a focus on adolescent and youth lived experiences of healthy eating and active living

### Adolescents and youth as citizen scientists and agents of change (WP2)

Behavioural risk factors with implications for adult health begin to emerge before and during adolescence. Therefore, adolescence is a critical window for the development of health promoting behaviours [12]. Almost nine out of 10 of the world’s 10 to 24-year olds live in less developed countries [13] with adolescents accounting for nearly one quarter of the population in sub-Saharan Africa and 1 in 5 of the population in Latin America and the Caribbean [13]. The GDAR Network has, therefore, focused on this critical window and built a research agenda around understanding and intervening to address the levers, putative determinants for diet and PA in adolescents at varying socio-ecological levels. Existing literature, predominantly from high-income countries has identified schools as a critical context for adolescent diet and activity behaviours [14–16]. Much of this literature has focused on primary school aged children, though adolescents may exercise greater volition than younger children over their health behaviours, while simultaneously being vulnerable to external influence such as advertising and social norms.

Research typically entails examining the environments within and immediately around schools [14], though students may be exposed to a much more diverse range of environments on the journey between home and school, particularly amongst those that travel long distances to attend school [17]. To respond to this knowledge gap, the GDAR Network research focused on the complex interaction between the sociocultural and environmental contexts at home, in school, as well as on the adolescents’ journeys to and from school. We believe this will elucidate how these urban exposures shape the diet and PA behaviours of adolescents within these communities (Fig. 1).

This work package employed mixed methods to capture the lived experiences. Quantitative approaches include objective and subjective measures of diet and PA. Much of the quantitative research methodology developed in other settings to measure nutrition and physical activity can be adapted to settings in the Global South. However, the context within which these health behaviours occur is critical to our understanding and potential to intervene. Capturing the lived experiences of people living in resource, and consequently choice, constrained environments, who face challenges that are very different to other more developed settings, is important in understanding health behaviours. The methods used to investigate the lived experience of participants extend from ethnography to in-depth interviews and focus group discussions. Research from the Global South has traditionally had to rely on innovative and pragmatic approaches to measuring and understanding diet and PA environments [18, 19]. Other quantitative approaches include researcher-obtained geolocation of the food and activity environments. Qualitative approaches deployed by GDAR include participatory photovoice methods that captures audio, visual and narrative data through a smart phone application [20] allowing the adolescent to map and describe environmental level factors that influence their diet and physical activity behaviours. The study of these levers incorporates both the researchers’ perspectives as well as that of the target population through the involvement of the adolescents as citizen scientists.

## Co-designing future interventions

To address upstream determinants, intersectoral action is required with these policies aligned for health creation. Multi-sectoral action, from health as well as non-health sectors including housing, transport and waste, and multi-stakeholder engagement, have been recognised as priorities for NCD prevention and control by the first Global Ministerial Conference on Healthy Lifestyles and Non-communicable Disease Control in 2011 and the World Health Organization (WHO) Global Action Plan on NCDs (2013–2020) [21]. A systematised review of the existing evidence exploring the association between informal settlement characteristics and health within South Africa highlighted the paucity of evidence to support the impact of intersectoral interventions and policies on health [22]. GDAR research is addressing this knowledge gap through natural experimental and modelling studies:

### Natural experimental study methods and food environments (WP4)

Over the past decade, a range of population-level dietary interventions has been systematically evaluated and identified as promising interventions to address dietary risk factors for NCDs [23]. These include, but are not limited to, mass media campaigns, food and menu labelling, fiscal pricing strategies (subsidies and taxation of foods and beverages), and food procurement policies in schools [23]. However, public policies with potential impact on upstream determinants of health are rarely implemented with rigorous evaluation designs. Unlike certain public health interventions (e.g. measles vaccination), the health impact of structural interventions (e.g. building a new grocery store) cannot be evaluated through the gold standard randomized control trials [24]. Natural experimental studies have been recommended as an alternative method to measure the impact of population-level policies [25]. This method is considered appropriate when random allocation of the intervention is not feasible, and when the researcher does not have control over the intervention [25].

Population level interventions are rarely accompanied by monitoring plans, thereby hindering evaluation efforts to measure their effectiveness. Therefore, knowledge gaps remain about whether, for whom and how these strategies impact dietary intake and disease outcomes; and the cost-effectiveness of such strategies. The GDAR Network researchers aim to address this knowledge gap and generate evidence utilising natural experimental designs, an approach that takes the opportunity of an intervention to understand the impact of population-level policies on health outcomes [25]. Two GDAR studies, one in Kenya and one in South Africa, applied this method to the evaluation of food environment interventions.

The work in Kenya (WP4a) revolved around a new hypermarket (a complex with diverse outlets including a supermarket, department stores, and eateries) under construction at Lake Basin Mall in Kisumu. The hypermarket is situated between a developing, high and middle socio-economic status residential area and a slum area close by. It was anticipated that the hypermarket would impact where local people buy food and consequently their dietary behaviours, and potentially anthropometric outcomes and overall nutritional status in the local population. Accordingly, the GDAR Network research agenda included a work package to investigate baseline dietary behaviours in Kisumu and in a comparison area (Homabay) in order to document the changing food environment in a rapidly urbanising settings, and to evaluate the impact of a new supermarket in Kisumu on community member’s dietary behaviours and health risk profiles, comparing these changes to dietary patterns in Homabay where there were no planned large food environment interventions.

The intervention evaluated in South Africa was a voluntary pledge by Coca-Cola to remove its products and advertising from primary schools (WP4b). This voluntary pledge emerged alongside government-led initiatives, in particular the taxation of sugary-sweetened beverages (SSBs), to support the implementation of the national NCD and obesity strategic plans [26, 27]. In 2017, Coca-Cola Beverages South Africa announced in a letter to principals of primary schools in Gauteng province that it would no longer supply school outlets with SSBs and would remove product and brand advertisements. While this study did not have a comparison area due to the province-wide nature of the intervention, the GDAR network nonetheless sought to investigate the degree to which this voluntary pledge had been implemented and whether it changed exposure to SSBs in schools in Gauteng province of South Africa.

By assessing the real time impact of interventions (beyond the control of the research team) being implemented in the food environment, the GDAR Network aims to contribute to the field of food environment research in African and Caribbean countries and to inform policies for NCD prevention (Fig. 1).

### Assessing availability of data to model health impact of transport policies (WP1b)

Many cities in Africa and the Caribbean have experienced rapid growth in cars and motorbikes, and some of the cities have high traffic fatality rates and air pollution levels. While cycling levels are low, walking continues to make up a large percentage of trips [28]. To understand how travel-related PA is likely to change and how active travel could be maintained and increased, it is important to consider the backdrop of road injuries and air pollution. The risk of injury (and intentional interpersonal violence) and impact of pollution can act as a deterrent to walking and cycling. However, although this broad pattern of change is often recited, we know little about variation between cities in how people travel, how this is changing, and the risks that people face.

Against a backdrop of rapid urbanisation, the specifics of how city level travel and road injuries and risk are changing for different population groups and the impact of transport and planning policies places are largely unknown. To develop evidence informed policies to improve population health by maintaining and increasing PA and reducing air pollution and traffic injuries, data are needed on what is happening now, and models are needed to estimate how changes in transport might affect health burdens. However, in many LMIC settings there is only limited surveillance of travel-related injury and poor understanding of how people move in the city and the associated injury risk.

To address the need for these data and health impact models in order to understand the health implications of changes in travel patterns, the GDAR Network undertook a scoping of available data in Africa and the Caribbean to inform development of contextual models. Data were identified and collected in Cape Town and Accra and are informing the refinement of a public health model initially developed for use in India and Latin America [29, 30], with the aim of further refining the model through additional data collection on household travel surveys and injury data in other African cities. In a collaboration with the WHO, we engaged with local stakeholders in Accra to access, appraise, and analyse data; and co-develop scenarios of possible futures. These were presented at a workshop in 2018 (https://sites.google.com/view/transportcam2019/home). By simultaneously engaging with policymakers in these countries through knowledge exchange sessions and policy roundtables, the research aims to provide evidence to better understand the health impact of intersectoral policies that influence physical activity (Fig. 1).

## Lessons learned to date

The GDAR Network aims for mutually beneficial collaboration to alleviate current NCD burdens and to provide scientific opportunities to drive stronger, and more sustainable science globally. Many research partnerships have been built on vital frameworks of core concepts and attributes [31–34] that ensure success and sustainability. To date, our Network’s research conducted in African and Caribbean countries has learned five key lessons aligned with some of these concepts and attributes, that may benefit other global health partnerships with similar structures and goals.

### LMIC co-investigators and HIC partners should co-create the research agenda

The GDAR Network has developed with a dual model of projects that involve all partners plus projects that are limited to only one site (such as the evaluation of the ‘coke school pledge’ in Johannesburg, and the new hypermarket in Kisumu) but from which all partners will learn. This dual model approach acknowledges and engages with the expertise that each partner possesses. No one partner dominates, and no one partner has the breadth or depth of expertise that exists across the network. Collectively partners across the network are able to support and feel ownership of a coordinated, interdisciplinary, research agenda rather than simply contributing to a series of independent projects. Building this type of network, with joint leadership and ownership of projects [33], is dependent on partners feeling able to communicate honesty and openly [32], perhaps most particularly when things are not going to plan.

An important component includes the knowledge generated for public health research investments from the South, particularly at the planning phase of the research. Limitations in the resources that are available to conduct research in LMICs, often force investigators and institutions to focus on “doing much with little”. Therefore, work undertaken in such settings highlights priority research areas, and directs attention to public health interventions that have been shown to save money and are cost-effective in comparison to other health care interventions. As with similar partnership initiatives [33], bidirectional knowledge exchange has been beneficial for Global North partners in the GDAR Network in terms of exposure to and experience with a diverse population of health needs and in terms of exposure to the global health determinants.

### LMIC institutions and personnel must explicitly benefit from capacity strengthening

The training of junior researchers is a key priority of the GDAR Network. Prior work has highlighted the professional advantages that members of networks like GDAR experience with the development of new skills or new ways of applying existing skills [35]. In the GDAR Network, a number of Master and PhD students are being trained and bespoke virtual training, led by different (Global South and North) partners is being developed that is relevant to the specific aspects of research that individuals are working on. As a result, being part of the network offers an opportunity for shared (South-South and North-South) learning. For example, partners in Jamaica have led cross-network training on qualitative in-depth interview methods, Cape Town partners on citizen-science methods, while Cambridge partners have facilitated training on natural experimental and evidence synthesis methods as well as in-person training on research finance administration for financial managers at all partner institutions.

The GDAR Network contributes to increasing the visibility and credibility of all partnering institutions and staff. A key future focus will be to effect a transition to continued and sustainable coordination among members of the GDAR network after the initial funding period.

### Engagement of non-academic partners to co-create the research priorities and approaches is essential

A successful approach that the GDAR Network has used is involving non-academic partners as core resources for co-creating research and the research agenda. The investigators identified the initial key partners and approached them to identify others. In this way the most important non-academic partners that had a bearing on this research project were approached and were involved in the effort of co-creating the research. At the partner country level, the partners engaged with government and other administrative structures that had a bearing on the project such as local and national government administrators, community elders, school leaders, local business people, NGO in the field of health and community development and any entities that would help further the GDAR agenda in the local community. For example, for the Kisumu hypermarket natural experimental study, these partners included officers from agricultural, trade, health, and faith-based organizations.

Communities were also engaged and informed of the research focus and objectives and consulted on the best ways to achieve the targets identified. In this way, an environment of goodwill and camaraderie was created from the beginning of interactions with the communities involved in the research. Tapping into the synergy created when an investigative team invites the community into the research process likely improves its success, since this success itself retains a definition shared by both the investigative team and the community. Such a process is important for NCD prevention interventions due to the need to understand and incorporate the culture of the community. As aspects of built and food environments are associated with physical activity, healthy eating and obesity, involving the community in the research process helps to build their capacity to understand and manage NCD prevention strategies [36]. Endeavouring to build the capacity of participating populations at multiple community levels lays a foundation for sustained impact on diet and physical activity transitions in LMICs, building on existing health and health determinant infrastructure [37–39]. This approach ensures the solicitation of the perspectives of community representatives on the best ways to achieve the objectives of the study and incorporation of those perspectives in the study design. Additionally, GDAR partners recruited data collectors for the project from the local community. For example, in Kenyan counties with high rates of youth unemployment, recruiting the data collectors from among the large pool of qualified youth worked to create employment and capacity building opportunities.

### Importance of the research funding landscape

The development of the GDAR network and research agenda was greatly facilitated by a new stream of UK funding, the overarching goal of which is to promote health, social and economic development in LMICs. Funding calls from this stream have emphasised the need to demonstrate that the proposed research is relevant to policy needs in partner LMICs and have also encouraged co-creation of research agenda with partners in LMICs. In applying for the main source of funding for this work, we were able to build in a period and process of joint research agenda setting (Fig. 2). Without this flexibility from the funder, it would have been much more difficult to jointly develop the research agenda or to build trust and equitable governance structures between all the partners. The initial funding was for 3 years, and while this is long enough to establish the network and implement the initial research agenda that we have described here, continued funding will be key to its longer-term impact. The timing of funding has been previously identified as a challenge to sustainability with the short length of funding streams (usually 3–5 years) and short-term government budgetary cycles and rapid turnover of ministers and CEOs a critical challenge to long-term global health research [31].

The development of GDAR has also been supported by a source of funds specifically to help build capacity for the financial management of research projects in LMICs. The need for developing capacity in this area is as relevant to the UK institution as to the LMIC institutions. Work in this area has included bringing together research project managers and financial administrators from all the institutions to learn together the strengths and challenges of financial management in each setting. An important part of this process is learning about the bureaucratic and research governance requirements of each institution such as the challenges of establishing collaboration agreements, ethical approval procedures and compliance with new data management regulations, in particular the European General Data Protection Regulation. This not only helps to facilitate more efficient setting up of agreements but also builds understanding and trust. While the nature of this particular funding stream was sufficiently flexible allowing a research agenda to be developed after the grant was awarded, the inherent power imbalance due to significant funding for global health from the global North poses a potential threat to the viability of this equitable approach. There is an increased appreciation of the value of co-created knowledge with research agendas and conduct driven by all partners and in consultation with stakeholders (Fig. 2). However this work can often only take place after funding is awarded. This highlights the importance of funding that facilitates the formation of research networks and the co-design of research between partners after the funding is awarded.

### Importance of incorporating an evaluation of the working of the network

Evaluation is a critical part of understanding and developing promising practice on partnerships [31], with existing resources available on indicators and frameworks for evaluating these partnerships [34]. We will be commissioning an independent formative evaluation designed to help the network partners understand how well the network is functioning and to highlight areas where we might do things differently. These areas will include governance, research agenda setting and implementation, capacity building, engagement with policy makers and other stakeholders. We will also investigate whether the network is functioning as more than the sum of its parts through sharing expertise and (South-North and South-South) learning across sites. We will consider the vision for the future development of the network, including its geographical and institutional scope, research agenda, and its ability to inform national, regional and international policy over the next 5 to 10 years. This independent evaluation is designed to ensure that the network will learn lessons that will support its further development and expansion to other partners, in order to deliver policy relevant research aimed at the prevention of non-communicable diseases.

## Conclusion

There is a clear case and urgent need for high quality policy-relevant research on diet and PA in Africa and the Caribbean. In this paper, we have set out our processes in developing the research agenda and experiences of working to foster shared ownership for sustainable capacity building and cutting edge policy-relevant research through the GDAR network. In this, we address current challenges building on opportunities and existing expertise to develop an innovative complementarity of methods to address complex population health challenges and inequities in the global health research partnership landscape. The Network is being utilised as a vehicle to enable researchers to exchange best practices in the areas of health promotion, legislation, regulation, and to share experience of the dissemination of scientific evidence related to common food and activity environment challenges for policy impact.

## Data Availability

Not applicable.

## Abbreviations

GDAR: Global Diet and Activity Research
HIC: High-Income Countries
LMIC: Low- and Middle-Income Countries
MRC: Medical Research Council
NCD: Non-Communicable Diseases
NIHR: National Institute for Health Research
PA: Physical Activity
SDG: Sustainable Development Goals
SSB: Sugar-Sweetened Beverages
WHO: World Health Organization
WP: Work Package
